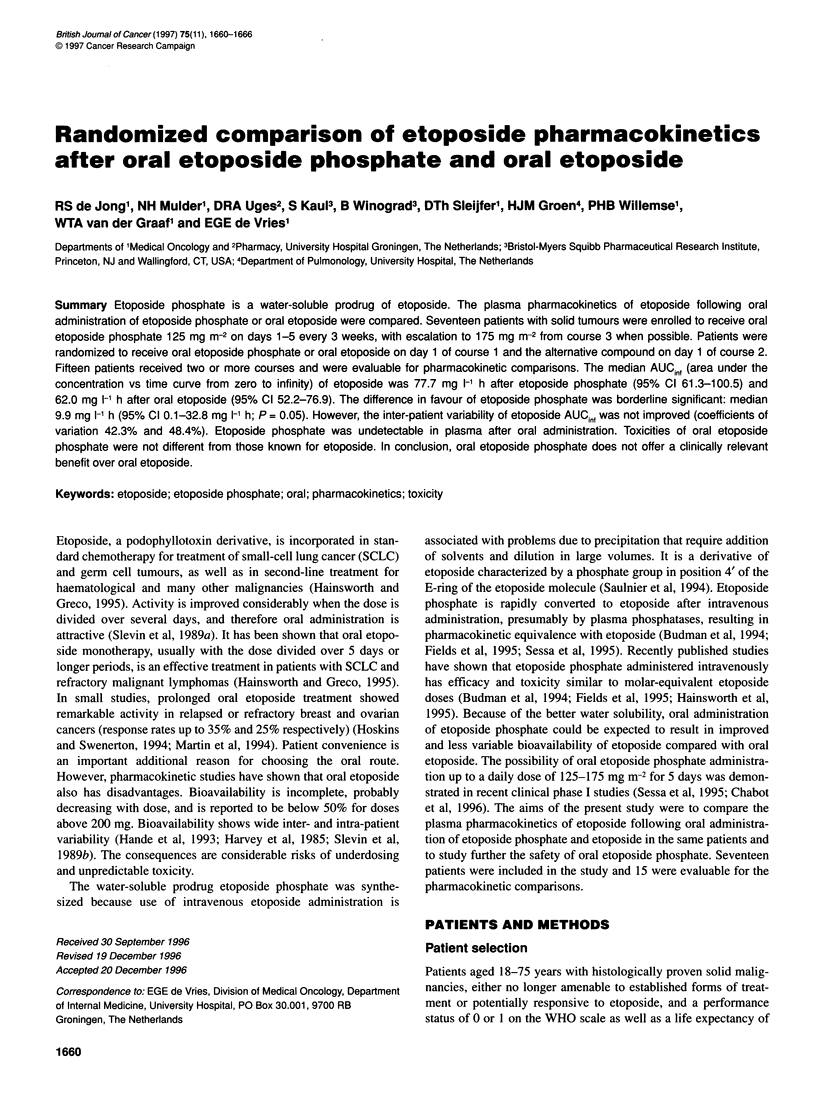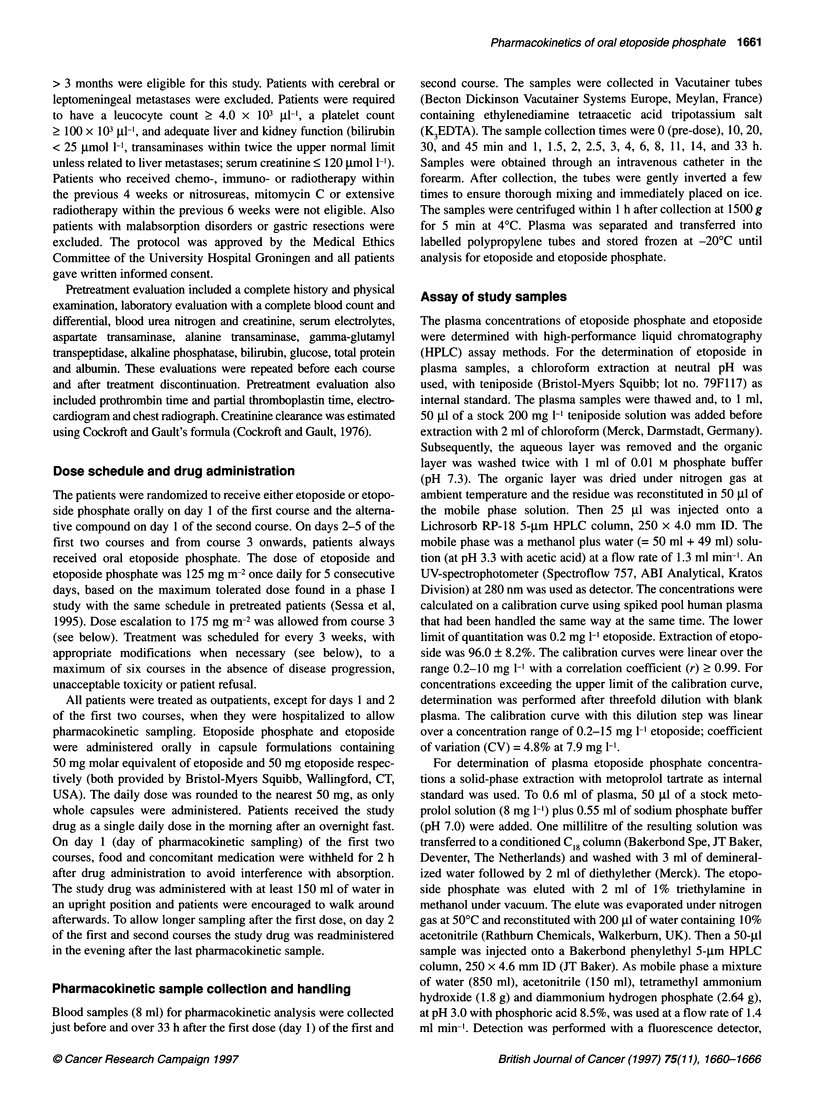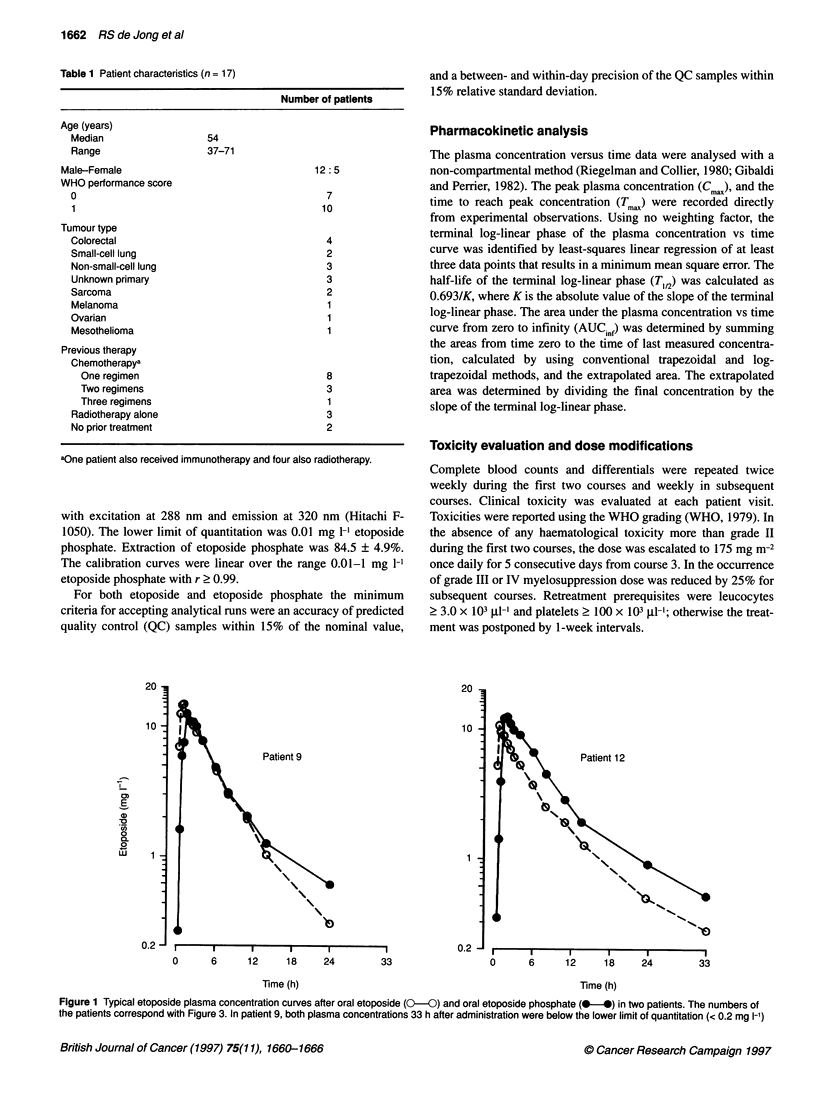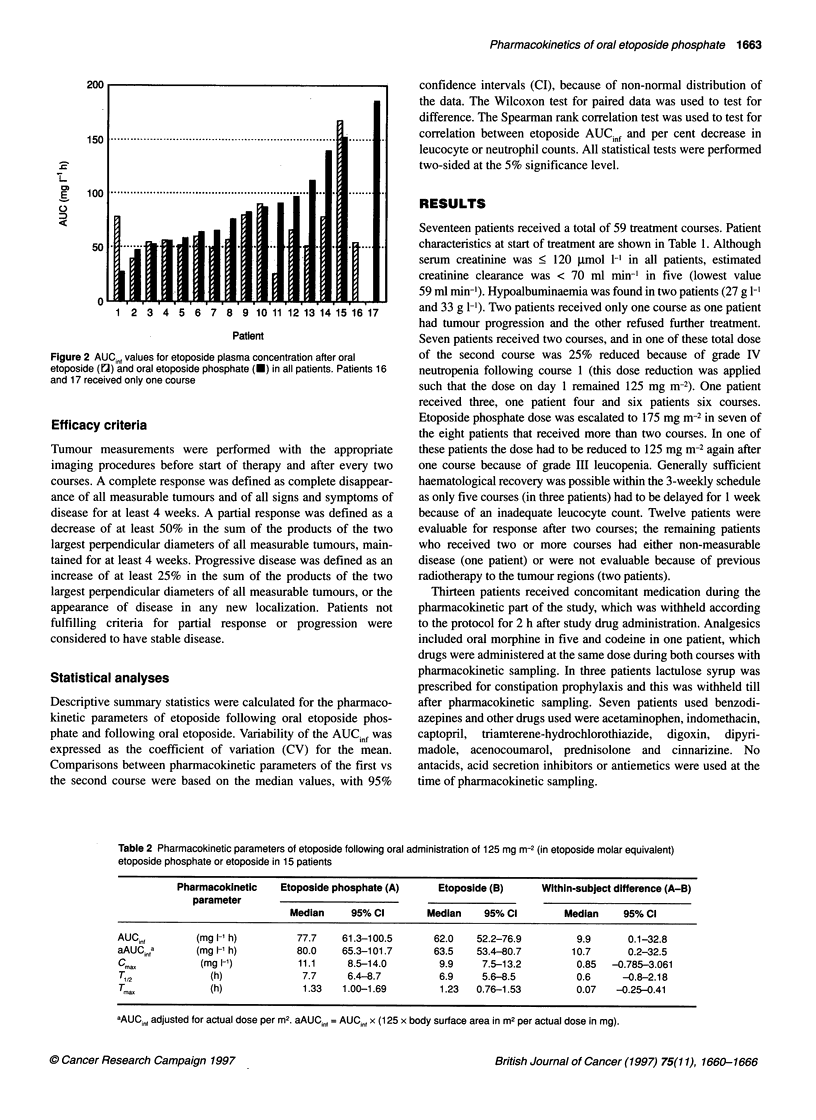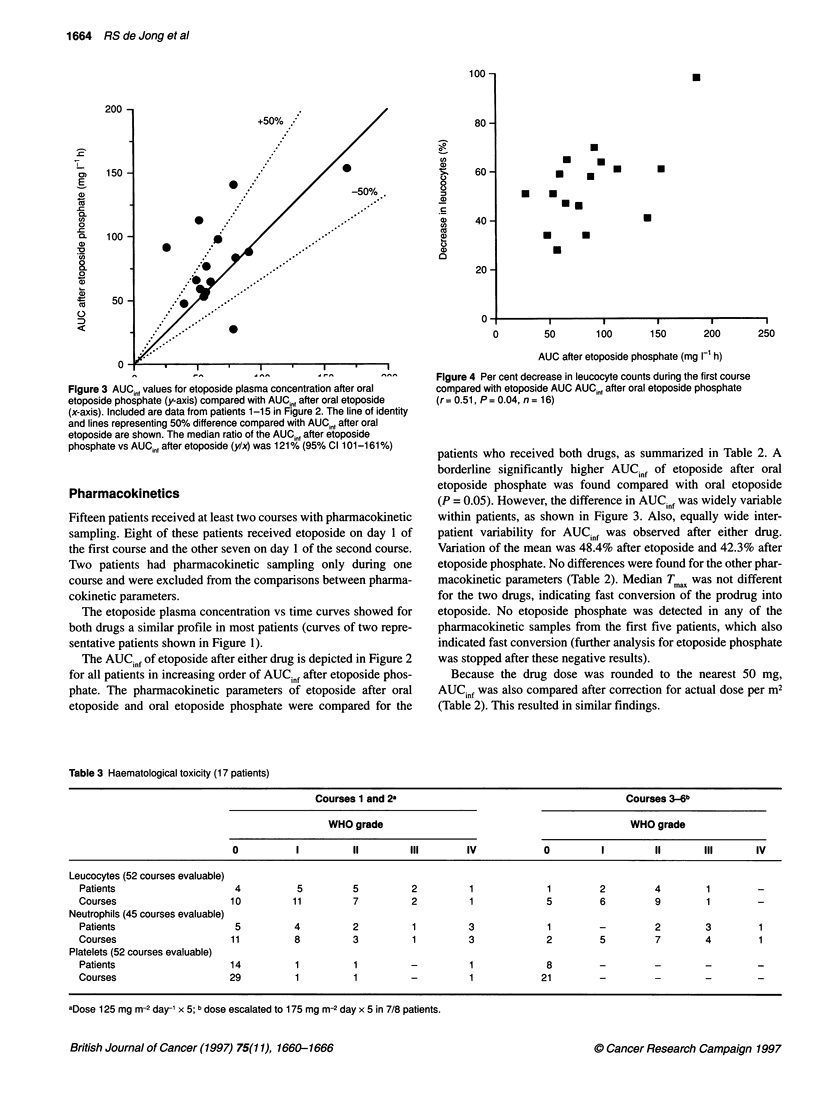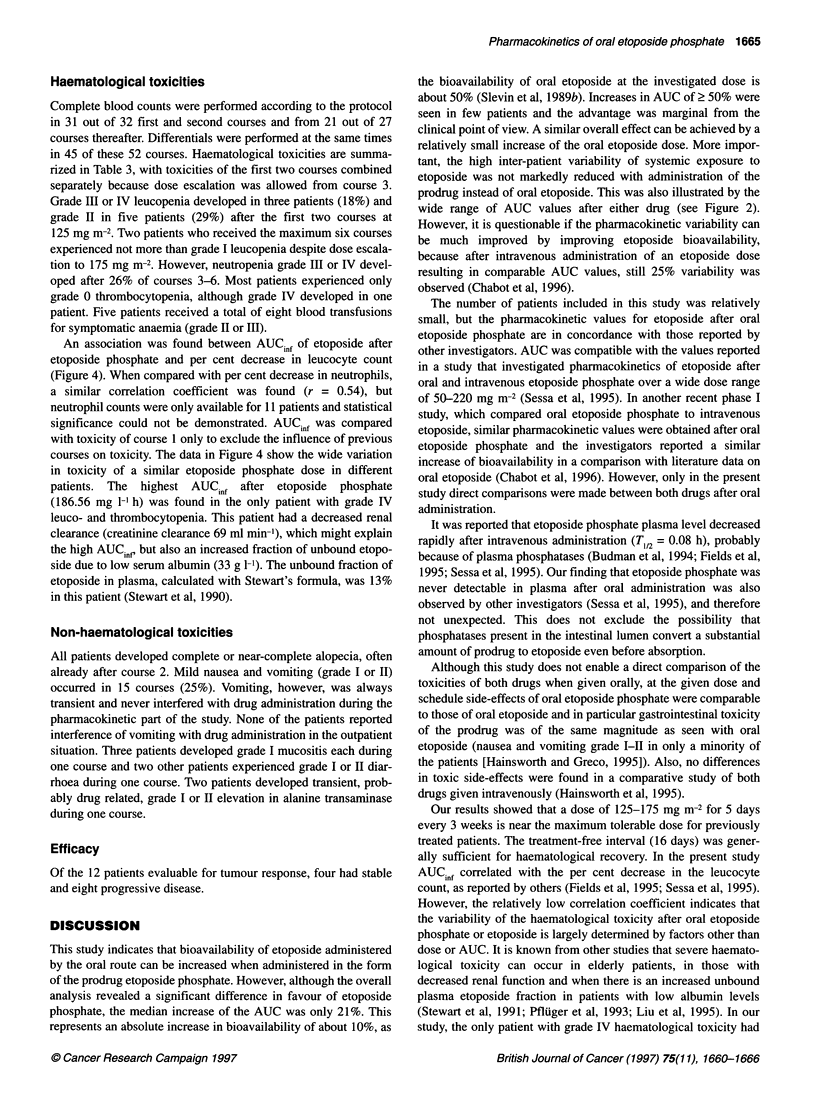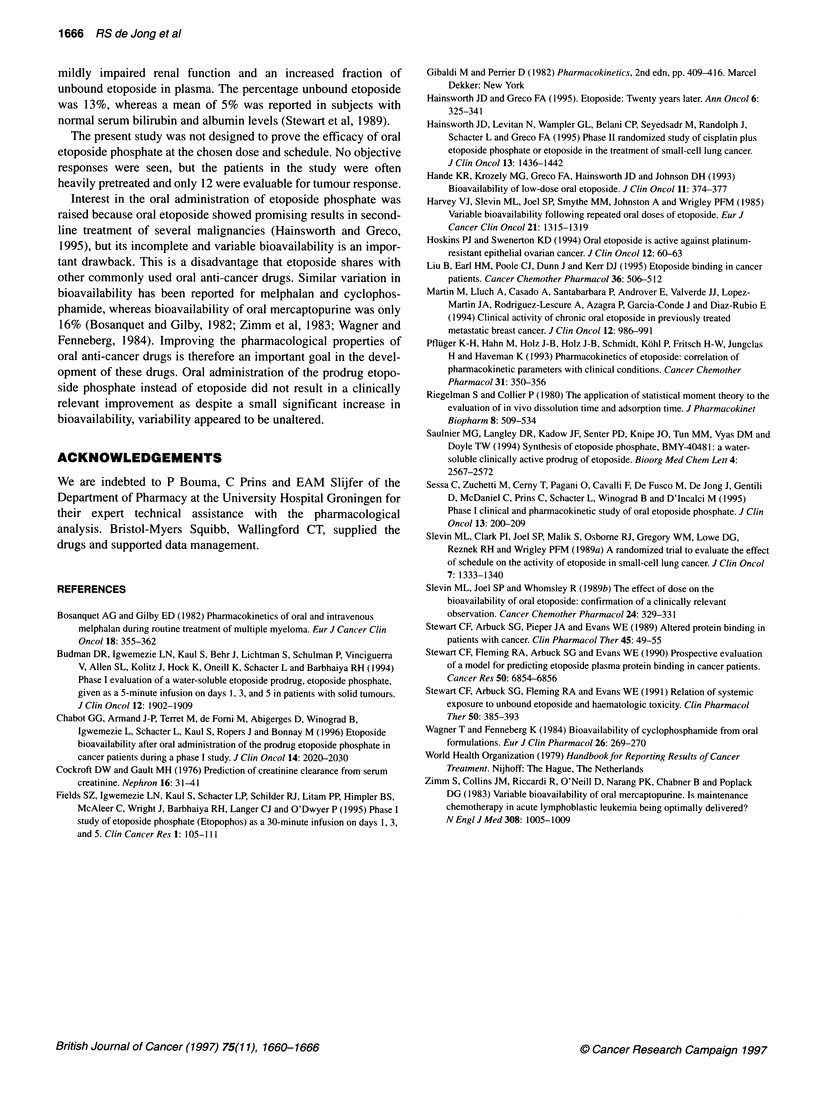# Randomized comparison of etoposide pharmacokinetics after oral etoposide phosphate and oral etoposide.

**DOI:** 10.1038/bjc.1997.282

**Published:** 1997

**Authors:** R. S. de Jong, N. H. Mulder, D. R. Uges, S. Kaul, B. Winograd, H. J. Groen, P. H. Willemse, W. T. van der Graaf, E. G. de Vries

**Affiliations:** Department of Medical Oncology, University Hospital Groningen, The Netherlands.

## Abstract

Etoposide phosphate is a water-soluble prodrug of etoposide. The plasma pharmacokinetics of etoposide following oral administration of etoposide phosphate or oral etoposide were compared. Seventeen patients with solid tumours were enrolled to receive oral etoposide phosphate 125 mg m(-2) on days 1-5 every 3 weeks, with escalation to 175 mg m(-2) from course 3 when possible. Patients were randomized to receive oral etoposide phosphate or oral etoposide on day 1 of course 1 and the alternative compound on day 1 of course 2. Fifteen patients received two or more courses and were evaluable for pharmacokinetic comparisons. The median AUC(inf) (area under the concentration vs time curve from zero to infinity) of etoposide was 77.7 mg l(-1) h after etoposide phosphate (95% CI 61.3-100.5) and 62.0 mg l(-1) h after oral etoposide (95% CI 52.2-76.9). The difference in favour of etoposide phosphate was borderline significant: median 9.9 mg l(-1) h (95% CI 0.1-32.8 mg l(-1) h; P = 0.05). However, the inter-patient variability of etoposide AUC(inf) was not improved (coefficients of variation 42.3% and 48.4%). Etoposide phosphate was undetectable in plasma after oral administration. Toxicities of oral etoposide phosphate were not different from those known for etoposide. In conclusion, oral etoposide phosphate does not offer a clinically relevant benefit over oral etoposide.


British Journal of Cancer (1997) 75(11), 1660–1666
© 1997 Cancer Research Campaign

### Randomized comparison of etoposide pharmacokinetics after oral etoposide phosphate and oral etoposide

**RS de Jong[1], NH Mulder[1], DRA Uges[2], S Kaul[3], B Winograd[3], DTh Sleijfer[1], HJM Groen[4], PHB Willemse[1], WTA van der Graaf[1] and EGE de Vries[1]**

Departments of [1]Medical Oncology and [2]Pharmacy, University Hospital Groningen, The Netherlands; [3]Bristol-Myers Squibb Pharmaceutical Research Institute, Princeton, NJ and Wallingford, CT, USA; [4]Department of Pulmonology, University Hospital, The Netherlands

**Summary** Etoposide phosphate is a water-soluble prodrug of etoposide. The plasma pharmacokinetics of etoposide following oral administration of etoposide phosphate or oral etoposide were compared. Seventeen patients with solid tumours were enrolled to receive oral etoposide phosphate 125 mg $m^{-2}$ on days 1–5 every 3 weeks, with escalation to 175 mg $m^{-2}$ from course 3 when possible. Patients were randomized to receive oral etoposide phosphate or oral etoposide on day 1 of course 1 and the alternative compound on day 1 of course 2. Fifteen patients received two or more courses and were evaluable for pharmacokinetic comparisons. The median $AUC_{inf}$ (area under the concentration vs time curve from zero to infinity) of etoposide was 77.7 mg $l^{-1}$ h after etoposide phosphate (95% CI 61.3–100.5) and 62.0 mg $l^{-1}$ h after oral etoposide (95% CI 52.2–76.9). The difference in favour of etoposide phosphate was borderline significant: median 9.9 mg $l^{-1}$ h (95% CI 0.1–32.8 mg $l^{-1}$ h; $P = 0.05$). However, the inter-patient variability of etoposide $AUC_{inf}$ was not improved (coefficients of variation 42.3% and 48.4%). Etoposide phosphate was undetectable in plasma after oral administration. Toxicities of oral etoposide phosphate were not different from those known for etoposide. In conclusion, oral etoposide phosphate does not offer a clinically relevant benefit over oral etoposide.

**Keywords:** etoposide; etoposide phosphate; oral; pharmacokinetics; toxicity

Etoposide, a podophyllotoxin derivative, is incorporated in standard chemotherapy for treatment of small-cell lung cancer (SCLC) and germ cell tumours, as well as in second-line treatment for haematological and many other malignancies (Hainsworth and Greco, 1995). Activity is improved considerably when the dose is divided over several days, and therefore oral administration is attractive (Slevin et al, 1989*a*). It has been shown that oral etoposide monotherapy, usually with the dose divided over 5 days or longer periods, is an effective treatment in patients with SCLC and refractory malignant lymphomas (Hainsworth and Greco, 1995). In small studies, prolonged oral etoposide treatment showed remarkable activity in relapsed or refractory breast and ovarian cancers (response rates up to 35% and 25% respectively) (Hoskins and Swenerton, 1994; Martin et al, 1994). Patient convenience is an important additional reason for choosing the oral route. However, pharmacokinetic studies have shown that oral etoposide also has disadvantages. Bioavailability is incomplete, probably decreasing with dose, and is reported to be below 50% for doses above 200 mg. Bioavailability shows wide inter- and intra-patient variability (Hande et al, 1993; Harvey et al, 1985; Slevin et al, 1989*b*). The consequences are considerable risks of underdosing and unpredictable toxicity.

The water-soluble prodrug etoposide phosphate was synthesized because use of intravenous etoposide administration is associated with problems due to precipitation that require addition of solvents and dilution in large volumes. It is a derivative of etoposide characterized by a phosphate group in position 4′ of the E-ring of the etoposide molecule (Saulnier et al, 1994). Etoposide phosphate is rapidly converted to etoposide after intravenous administration, presumably by plasma phosphatases, resulting in pharmacokinetic equivalence with etoposide (Budman et al, 1994; Fields et al, 1995; Sessa et al, 1995). Recently published studies have shown that etoposide phosphate administered intravenously has efficacy and toxicity similar to molar-equivalent etoposide doses (Budman et al, 1994; Fields et al, 1995; Hainsworth et al, 1995). Because of the better water solubility, oral administration of etoposide phosphate could be expected to result in improved and less variable bioavailability of etoposide compared with oral etoposide. The possibility of oral etoposide phosphate administration up to a daily dose of 125–175 mg $m^{-2}$ for 5 days was demonstrated in recent clinical phase I studies (Sessa et al, 1995; Chabot et al, 1996). The aims of the present study were to compare the plasma pharmacokinetics of etoposide following oral administration of etoposide phosphate and etoposide in the same patients and to study further the safety of oral etoposide phosphate. Seventeen patients were included in the study and 15 were evaluable for the pharmacokinetic comparisons.

*Received 30 September 1996*
*Revised 19 December 1996*
*Accepted 20 December 1996*

*Correspondence to:* EGE de Vries, Division of Medical Oncology, Department of Internal Medicine, University Hospital, PO Box 30.001, 9700 RB Groningen, The Netherlands

### PATIENTS AND METHODS

### Patient selection

Patients aged 18–75 years with histologically proven solid malignancies, either no longer amenable to established forms of treatment or potentially responsive to etoposide, and a performance status of 0 or 1 on the WHO scale as well as a life expectancy of

> 3 months were eligible for this study. Patients with cerebral or leptomeningeal metastases were excluded. Patients were required to have a leucocyte count ≥ 4.0 × 10³ μl⁻¹, a platelet count ≥ 100 × 10³ μl⁻¹, and adequate liver and kidney function (bilirubin < 25 μmol l⁻¹, transaminases within twice the upper normal limit unless related to liver metastases; serum creatinine ≤ 120 μmol l⁻¹). Patients who received chemo-, immuno- or radiotherapy within the previous 4 weeks or nitrosureas, mitomycin C or extensive radiotherapy within the previous 6 weeks were not eligible. Also patients with malabsorption disorders or gastric resections were excluded. The protocol was approved by the Medical Ethics Committee of the University Hospital Groningen and all patients gave written informed consent.

Pretreatment evaluation included a complete history and physical examination, laboratory evaluation with a complete blood count and differential, blood urea nitrogen and creatinine, serum electrolytes, aspartate transaminase, alanine transaminase, gamma-glutamyl transpeptidase, alkaline phosphatase, bilirubin, glucose, total protein and albumin. These evaluations were repeated before each course and after treatment discontinuation. Pretreatment evaluation also included prothrombin time and partial thromboplastin time, electrocardiogram and chest radiograph. Creatinine clearance was estimated using Cockroft and Gault's formula (Cockroft and Gault, 1976).

### Dose schedule and drug administration

The patients were randomized to receive either etoposide or etoposide phosphate orally on day 1 of the first course and the alternative compound on day 1 of the second course. On days 2–5 of the first two courses and from course 3 onwards, patients always received oral etoposide phosphate. The dose of etoposide and etoposide phosphate was 125 mg m⁻² once daily for 5 consecutive days, based on the maximum tolerated dose found in a phase I study with the same schedule in pretreated patients (Sessa et al, 1995). Dose escalation to 175 mg m⁻² was allowed from course 3 (see below). Treatment was scheduled for every 3 weeks, with appropriate modifications when necessary (see below), to a maximum of six courses in the absence of disease progression, unacceptable toxicity or patient refusal.

All patients were treated as outpatients, except for days 1 and 2 of the first two courses, when they were hospitalized to allow pharmacokinetic sampling. Etoposide phosphate and etoposide were administered orally in capsule formulations containing 50 mg molar equivalent of etoposide and 50 mg etoposide respectively (both provided by Bristol-Myers Squibb, Wallingford, CT, USA). The daily dose was rounded to the nearest 50 mg, as only whole capsules were administered. Patients received the study drug as a single daily dose in the morning after an overnight fast. On day 1 (day of pharmacokinetic sampling) of the first two courses, food and concomitant medication were withheld for 2 h after drug administration to avoid interference with absorption. The study drug was administered with at least 150 ml of water in an upright position and patients were encouraged to walk around afterwards. To allow longer sampling after the first dose, on day 2 of the first and second courses the study drug was readministered in the evening after the last pharmacokinetic sample.

### Pharmacokinetic sample collection and handling

Blood samples (8 ml) for pharmacokinetic analysis were collected just before and over 33 h after the first dose (day 1) of the first and second course. The samples were collected in Vacutainer tubes (Becton Dickinson Vacutainer Systems Europe, Meylan, France) containing ethylenediamine tetraacetic acid tripotassium salt ($K_3EDTA$). The sample collection times were 0 (pre-dose), 10, 20, 30, and 45 min and 1, 1.5, 2, 2.5, 3, 4, 6, 8, 11, 14, and 33 h. Samples were obtained through an intravenous catheter in the forearm. After collection, the tubes were gently inverted a few times to ensure thorough mixing and immediately placed on ice. The samples were centrifuged within 1 h after collection at 1500 ***g*** for 5 min at 4°C. Plasma was separated and transferred into labelled polypropylene tubes and stored frozen at –20°C until analysis for etoposide and etoposide phosphate.

### Assay of study samples

The plasma concentrations of etoposide phosphate and etoposide were determined with high-performance liquid chromatography (HPLC) assay methods. For the determination of etoposide in plasma samples, a chloroform extraction at neutral pH was used, with teniposide (Bristol-Myers Squibb; lot no. 79F117) as internal standard. The plasma samples were thawed and, to 1 ml, 50 μl of a stock 200 mg l⁻¹ teniposide solution was added before extraction with 2 ml of chloroform (Merck, Darmstadt, Germany). Subsequently, the aqueous layer was removed and the organic layer was washed twice with 1 ml of 0.01 M phosphate buffer (pH 7.3). The organic layer was dried under nitrogen gas at ambient temperature and the residue was reconstituted in 50 μl of the mobile phase solution. Then 25 μl was injected onto a Lichrosorb RP-18 5-μm HPLC column, 250 × 4.0 mm ID. The mobile phase was a methanol plus water (= 50 ml + 49 ml) solution (at pH 3.3 with acetic acid) at a flow rate of 1.3 ml min⁻¹. An UV-spectrophotometer (Spectroflow 757, ABI Analytical, Kratos Division) at 280 nm was used as detector. The concentrations were calculated on a calibration curve using spiked pool human plasma that had been handled the same way at the same time. The lower limit of quantitation was 0.2 mg l⁻¹ etoposide. Extraction of etoposide was 96.0 ± 8.2%. The calibration curves were linear over the range 0.2–10 mg l⁻¹ with a correlation coefficient ($r$) ≥ 0.99. For concentrations exceeding the upper limit of the calibration curve, determination was performed after threefold dilution with blank plasma. The calibration curve with this dilution step was linear over a concentration range of 0.2–15 mg l⁻¹ etoposide; coefficient of variation (CV) = 4.8% at 7.9 mg l⁻¹.

For determination of plasma etoposide phosphate concentrations a solid-phase extraction with metoprolol tartrate as internal standard was used. To 0.6 ml of plasma, 50 μl of a stock metoprolol solution (8 mg l⁻¹) plus 0.55 ml of sodium phosphate buffer (pH 7.0) were added. One millilitre of the resulting solution was transferred to a conditioned $C_{18}$ column (Bakerbond Spe, JT Baker, Deventer, The Netherlands) and washed with 3 ml of demineralized water followed by 2 ml of diethylether (Merck). The etoposide phosphate was eluted with 2 ml of 1% triethylamine in methanol under vacuum. The elute was evaporated under nitrogen gas at 50°C and reconstituted with 200 μl of water containing 10% acetonitrile (Rathburn Chemicals, Walkerburn, UK). Then a 50-μl sample was injected onto a Bakerbond phenylethyl 5-μm HPLC column, 250 × 4.6 mm ID (JT Baker). As mobile phase a mixture of water (850 ml), acetonitrile (150 ml), tetramethyl ammonium hydroxide (1.8 g) and diammonium hydrogen phosphate (2.64 g), at pH 3.0 with phosphoric acid 8.5%, was used at a flow rate of 1.4 ml min⁻¹. Detection was performed with a fluorescence detector,

© Cancer Research Campaign 1997

**Table 1** Patient characteristics ($n$ = 17)

| | | Number of patients |
|---|---|---|
| Age (years) | | |
| Median | 54 | |
| Range | 37–71 | |
| Male–Female | | 12 : 5 |
| WHO performance score | | |
| 0 | | 7 |
| 1 | | 10 |
| Tumour type | | |
| Colorectal | | 4 |
| Small-cell lung | | 2 |
| Non-small-cell lung | | 3 |
| Unknown primary | | 3 |
| Sarcoma | | 2 |
| Melanoma | | 1 |
| Ovarian | | 1 |
| Mesothelioma | | 1 |
| Previous therapy | | |
| Chemotherapy[a] | | |
| One regimen | | 8 |
| Two regimens | | 3 |
| Three regimens | | 1 |
| Radiotherapy alone | | 3 |
| No prior treatment | | 2 |

[a]One patient also received immunotherapy and four also radiotherapy.

with excitation at 288 nm and emission at 320 nm (Hitachi F-1050). The lower limit of quantitation was 0.01 mg $l^{-1}$ etoposide phosphate. Extraction of etoposide phosphate was 84.5 ± 4.9%. The calibration curves were linear over the range 0.01–1 mg $l^{-1}$ etoposide phosphate with $r \geq 0.99$.

For both etoposide and etoposide phosphate the minimum criteria for accepting analytical runs were an accuracy of predicted quality control (QC) samples within 15% of the nominal value, and a between- and within-day precision of the QC samples within 15% relative standard deviation.

### Pharmacokinetic analysis

The plasma concentration versus time data were analysed with a non-compartmental method (Riegelman and Collier, 1980; Gibaldi and Perrier, 1982). The peak plasma concentration ($C_{max}$), and the time to reach peak concentration ($T_{max}$) were recorded directly from experimental observations. Using no weighting factor, the terminal log-linear phase of the plasma concentration vs time curve was identified by least-squares linear regression of at least three data points that results in a minimum mean square error. The half-life of the terminal log-linear phase ($T_{1/2}$) was calculated as $0.693/K$, where $K$ is the absolute value of the slope of the terminal log-linear phase. The area under the plasma concentration vs time curve from zero to infinity ($AUC_{inf}$) was determined by summing the areas from time zero to the time of last measured concentration, calculated by using conventional trapezoidal and log-trapezoidal methods, and the extrapolated area. The extrapolated area was determined by dividing the final concentration by the slope of the terminal log-linear phase.

### Toxicity evaluation and dose modifications

Complete blood counts and differentials were repeated twice weekly during the first two courses and weekly in subsequent courses. Clinical toxicity was evaluated at each patient visit. Toxicities were reported using the WHO grading (WHO, 1979). In the absence of any haematological toxicity more than grade II during the first two courses, the dose was escalated to 175 mg $m^{-2}$ once daily for 5 consecutive days from course 3. In the occurrence of grade III or IV myelosuppression dose was reduced by 25% for subsequent courses. Retreatment prerequisites were leucocytes $\geq 3.0 \times 10^3\ \mu l^{-1}$ and platelets $\geq 100 \times 10^3\ \mu l^{-1}$; otherwise the treatment was postponed by 1-week intervals.

**Figure 1** Typical etoposide plasma concentration curves after oral etoposide (O—O) and oral etoposide phosphate (●—●) in two patients. The numbers of the patients correspond with Figure 3. In patient 9, both plasma concentrations 33 h after administration were below the lower limit of quantitation (< 0.2 mg $l^{-1}$)

© Cancer Research Campaign 1997

**Figure 2** $AUC_{inf}$ values for etoposide plasma concentration after oral etoposide (◪) and oral etoposide phosphate (■) in all patients. Patients 16 and 17 received only one course

### Efficacy criteria

Tumour measurements were performed with the appropriate imaging procedures before start of therapy and after every two courses. A complete response was defined as complete disappearance of all measurable tumours and of all signs and symptoms of disease for at least 4 weeks. A partial response was defined as a decrease of at least 50% in the sum of the products of the two largest perpendicular diameters of all measurable tumours, maintained for at least 4 weeks. Progressive disease was defined as an increase of at least 25% in the sum of the products of the two largest perpendicular diameters of all measurable tumours, or the appearance of disease in any new localization. Patients not fulfilling criteria for partial response or progression were considered to have stable disease.

### Statistical analyses

Descriptive summary statistics were calculated for the pharmacokinetic parameters of etoposide following oral etoposide phosphate and following oral etoposide. Variability of the $AUC_{inf}$ was expressed as the coefficient of variation (CV) for the mean. Comparisons between pharmacokinetic parameters of the first vs the second course were based on the median values, with 95% confidence intervals (CI), because of non-normal distribution of the data. The Wilcoxon test for paired data was used to test for difference. The Spearman rank correlation test was used to test for correlation between etoposide $AUC_{inf}$ and per cent decrease in leucocyte or neutrophil counts. All statistical tests were performed two-sided at the 5% significance level.

### RESULTS

Seventeen patients received a total of 59 treatment courses. Patient characteristics at start of treatment are shown in Table 1. Although serum creatinine was ≤ 120 μmol $l^{-1}$ in all patients, estimated creatinine clearance was < 70 ml $min^{-1}$ in five (lowest value 59 ml $min^{-1}$). Hypoalbuminaemia was found in two patients (27 g $l^{-1}$ and 33 g $l^{-1}$). Two patients received only one course as one patient had tumour progression and the other refused further treatment. Seven patients received two courses, and in one of these total dose of the second course was 25% reduced because of grade IV neutropenia following course 1 (this dose reduction was applied such that the dose on day 1 remained 125 mg $m^{-2}$). One patient received three, one patient four and six patients six courses. Etoposide phosphate dose was escalated to 175 mg $m^{-2}$ in seven of the eight patients that received more than two courses. In one of these patients the dose had to be reduced to 125 mg $m^{-2}$ again after one course because of grade III leucopenia. Generally sufficient haematological recovery was possible within the 3-weekly schedule as only five courses (in three patients) had to be delayed for 1 week because of an inadequate leucocyte count. Twelve patients were evaluable for response after two courses; the remaining patients who received two or more courses had either non-measurable disease (one patient) or were not evaluable because of previous radiotherapy to the tumour regions (two patients).

Thirteen patients received concomitant medication during the pharmacokinetic part of the study, which was withheld according to the protocol for 2 h after study drug administration. Analgesics included oral morphine in five and codeine in one patient, which drugs were administered at the same dose during both courses with pharmacokinetic sampling. In three patients lactulose syrup was prescribed for constipation prophylaxis and this was withheld till after pharmacokinetic sampling. Seven patients used benzodiazepines and other drugs used were acetaminophen, indomethacin, captopril, triamterene-hydrochlorothiazide, digoxin, dipyrimadole, acenocoumarol, prednisolone and cinnarizine. No antacids, acid secretion inhibitors or antiemetics were used at the time of pharmacokinetic sampling.

**Table 2** Pharmacokinetic parameters of etoposide following oral administration of 125 mg $m^{-2}$ (in etoposide molar equivalent) etoposide phosphate or etoposide in 15 patients

| | Pharmacokinetic parameter | Etoposide phosphate (A) | | Etoposide (B) | | Within-subject difference (A–B) | |
|---|---|---|---|---|---|---|---|
| | | Median | 95% CI | Median | 95% CI | Median | 95% CI |
| $AUC_{inf}$ | (mg $l^{-1}$ h) | 77.7 | 61.3–100.5 | 62.0 | 52.2–76.9 | 9.9 | 0.1–32.8 |
| $aAUC_{inf}$[a] | (mg $l^{-1}$ h) | 80.0 | 65.3–101.7 | 63.5 | 53.4–80.7 | 10.7 | 0.2–32.5 |
| $C_{max}$ | (mg $l^{-1}$) | 11.1 | 8.5–14.0 | 9.9 | 7.5–13.2 | 0.85 | –0.785–3.061 |
| $T_{1/2}$ | (h) | 7.7 | 6.4–8.7 | 6.9 | 5.6–8.5 | 0.6 | –0.8–2.18 |
| $T_{max}$ | (h) | 1.33 | 1.00–1.69 | 1.23 | 0.76–1.53 | 0.07 | –0.25–0.41 |

[a]$AUC_{inf}$ adjusted for actual dose per $m^2$. $aAUC_{inf} = AUC_{inf} \times$ (125 × body surface area in $m^2$ per actual dose in mg).

© Cancer Research Campaign 1997

**Figure 3** $AUC_{inf}$ values for etoposide plasma concentration after oral etoposide phosphate (*y*-axis) compared with $AUC_{inf}$ after oral etoposide (*x*-axis). Included are data from patients 1–15 in Figure 2. The line of identity and lines representing 50% difference compared with $AUC_{inf}$ after oral etoposide are shown. The median ratio of the $AUC_{inf}$ after etoposide phosphate vs $AUC_{inf}$ after etoposide (*y*/*x*) was 121% (95% CI 101–161%)

**Figure 4** Per cent decrease in leucocyte counts during the first course compared with etoposide AUC $AUC_{inf}$ after oral etoposide phosphate ($r$ = 0.51, $P$ = 0.04, $n$ = 16)

### Pharmacokinetics

Fifteen patients received at least two courses with pharmacokinetic sampling. Eight of these patients received etoposide on day 1 of the first course and the other seven on day 1 of the second course. Two patients had pharmacokinetic sampling only during one course and were excluded from the comparisons between pharmacokinetic parameters.

The etoposide plasma concentration vs time curves showed for both drugs a similar profile in most patients (curves of two representative patients shown in Figure 1).

The $AUC_{inf}$ of etoposide after either drug is depicted in Figure 2 for all patients in increasing order of $AUC_{inf}$ after etoposide phosphate. The pharmacokinetic parameters of etoposide after oral etoposide and oral etoposide phosphate were compared for the patients who received both drugs, as summarized in Table 2. A borderline significantly higher $AUC_{inf}$ of etoposide after oral etoposide phosphate was found compared with oral etoposide ($P = 0.05$). However, the difference in $AUC_{inf}$ was widely variable within patients, as shown in Figure 3. Also, equally wide inter-patient variability for $AUC_{inf}$ was observed after either drug. Variation of the mean was 48.4% after etoposide and 42.3% after etoposide phosphate. No differences were found for the other pharmacokinetic parameters (Table 2). Median $T_{max}$ was not different for the two drugs, indicating fast conversion of the prodrug into etoposide. No etoposide phosphate was detected in any of the pharmacokinetic samples from the first five patients, which also indicated fast conversion (further analysis for etoposide phosphate was stopped after these negative results).

Because the drug dose was rounded to the nearest 50 mg, $AUC_{inf}$ was also compared after correction for actual dose per $m^2$ (Table 2). This resulted in similar findings.

**Table 3** Haematological toxicity (17 patients)

| | Courses 1 and 2[a] | | | | | Courses 3–6[b] | | | | |
|---|---|---|---|---|---|---|---|---|---|---|
| | WHO grade | | | | | WHO grade | | | | |
| | 0 | I | II | III | IV | 0 | I | II | III | IV |
| Leucocytes (52 courses evaluable) | | | | | | | | | | |
| Patients | 4 | 5 | 5 | 2 | 1 | 1 | 2 | 4 | 1 | – |
| Courses | 10 | 11 | 7 | 2 | 1 | 5 | 6 | 9 | 1 | – |
| Neutrophils (45 courses evaluable) | | | | | | | | | | |
| Patients | 5 | 4 | 2 | 1 | 3 | 1 | – | 2 | 3 | 1 |
| Courses | 11 | 8 | 3 | 1 | 3 | 2 | 5 | 7 | 4 | 1 |
| Platelets (52 courses evaluable) | | | | | | | | | | |
| Patients | 14 | 1 | 1 | – | 1 | 8 | – | – | – | – |
| Courses | 29 | 1 | 1 | – | 1 | 21 | – | – | – | – |

[a]Dose 125 mg $m^{-2}$ $day^{-1}$ × 5; [b] dose escalated to 175 mg $m^{-2}$ day × 5 in 7/8 patients.

© Cancer Research Campaign 1997

### Haematological toxicities

Complete blood counts were performed according to the protocol in 31 out of 32 first and second courses and from 21 out of 27 courses thereafter. Differentials were performed at the same times in 45 of these 52 courses. Haematological toxicities are summarized in Table 3, with toxicities of the first two courses combined separately because dose escalation was allowed from course 3. Grade III or IV leucopenia developed in three patients (18%) and grade II in five patients (29%) after the first two courses at 125 mg $m^{-2}$. Two patients who received the maximum six courses experienced not more than grade I leucopenia despite dose escalation to 175 mg $m^{-2}$. However, neutropenia grade III or IV developed after 26% of courses 3–6. Most patients experienced only grade 0 thrombocytopenia, although grade IV developed in one patient. Five patients received a total of eight blood transfusions for symptomatic anaemia (grade II or III).

An association was found between $AUC_{inf}$ of etoposide after etoposide phosphate and per cent decrease in leucocyte count (Figure 4). When compared with per cent decrease in neutrophils, a similar correlation coefficient was found ($r$ = 0.54), but neutrophil counts were only available for 11 patients and statistical significance could not be demonstrated. $AUC_{inf}$ was compared with toxicity of course 1 only to exclude the influence of previous courses on toxicity. The data in Figure 4 show the wide variation in toxicity of a similar etoposide phosphate dose in different patients. The highest $AUC_{inf}$ after etoposide phosphate (186.56 mg $l^{-1}$ h) was found in the only patient with grade IV leuco- and thrombocytopenia. This patient had a decreased renal clearance (creatinine clearance 69 ml $min^{-1}$), which might explain the high $AUC_{inf}$, but also an increased fraction of unbound etoposide due to low serum albumin (33 g $l^{-1}$). The unbound fraction of etoposide in plasma, calculated with Stewart's formula, was 13% in this patient (Stewart et al, 1990).

### Non-haematological toxicities

All patients developed complete or near-complete alopecia, often already after course 2. Mild nausea and vomiting (grade I or II) occurred in 15 courses (25%). Vomiting, however, was always transient and never interfered with drug administration during the pharmacokinetic part of the study. None of the patients reported interference of vomiting with drug administration in the outpatient situation. Three patients developed grade I mucositis each during one course and two other patients experienced grade I or II diarrhoea during one course. Two patients developed transient, probably drug related, grade I or II elevation in alanine transaminase during one course.

### Efficacy

Of the 12 patients evaluable for tumour response, four had stable and eight progressive disease.

### DISCUSSION

This study indicates that bioavailability of etoposide administered by the oral route can be increased when administered in the form of the prodrug etoposide phosphate. However, although the overall analysis revealed a significant difference in favour of etoposide phosphate, the median increase of the AUC was only 21%. This represents an absolute increase in bioavailability of about 10%, as the bioavailability of oral etoposide at the investigated dose is about 50% (Slevin et al, 1989*b*). Increases in AUC of ≥ 50% were seen in few patients and the advantage was marginal from the clinical point of view. A similar overall effect can be achieved by a relatively small increase of the oral etoposide dose. More important, the high inter-patient variability of systemic exposure to etoposide was not markedly reduced with administration of the prodrug instead of oral etoposide. This was also illustrated by the wide range of AUC values after either drug (see Figure 2). However, it is questionable if the pharmacokinetic variability can be much improved by improving etoposide bioavailability, because after intravenous administration of an etoposide dose resulting in comparable AUC values, still 25% variability was observed (Chabot et al, 1996).

The number of patients included in this study was relatively small, but the pharmacokinetic values for etoposide after oral etoposide phosphate are in concordance with those reported by other investigators. AUC was compatible with the values reported in a study that investigated pharmacokinetics of etoposide after oral and intravenous etoposide phosphate over a wide dose range of 50–220 mg $m^{-2}$ (Sessa et al, 1995). In another recent phase I study, which compared oral etoposide phosphate to intravenous etoposide, similar pharmacokinetic values were obtained after oral etoposide phosphate and the investigators reported a similar increase of bioavailability in a comparison with literature data on oral etoposide (Chabot et al, 1996). However, only in the present study direct comparisons were made between both drugs after oral administration.

It was reported that etoposide phosphate plasma level decreased rapidly after intravenous administration ($T_{1/2}$ = 0.08 h), probably because of plasma phosphatases (Budman et al, 1994; Fields et al, 1995; Sessa et al, 1995). Our finding that etoposide phosphate was never detectable in plasma after oral administration was also observed by other investigators (Sessa et al, 1995), and therefore not unexpected. This does not exclude the possibility that phosphatases present in the intestinal lumen convert a substantial amount of prodrug to etoposide even before absorption.

Although this study does not enable a direct comparison of the toxicities of both drugs when given orally, at the given dose and schedule side-effects of oral etoposide phosphate were comparable to those of oral etoposide and in particular gastrointestinal toxicity of the prodrug was of the same magnitude as seen with oral etoposide (nausea and vomiting grade I–II in only a minority of the patients [Hainsworth and Greco, 1995]). Also, no differences in toxic side-effects were found in a comparative study of both drugs given intravenously (Hainsworth et al, 1995).

Our results showed that a dose of 125–175 mg $m^{-2}$ for 5 days every 3 weeks is near the maximum tolerable dose for previously treated patients. The treatment-free interval (16 days) was generally sufficient for haematological recovery. In the present study $AUC_{inf}$ correlated with the per cent decrease in the leucocyte count, as reported by others (Fields et al, 1995; Sessa et al, 1995). However, the relatively low correlation coefficient indicates that the variability of the haematological toxicity after oral etoposide phosphate or etoposide is largely determined by factors other than dose or AUC. It is known from other studies that severe haematological toxicity can occur in elderly patients, in those with decreased renal function and when there is an increased unbound plasma etoposide fraction in patients with low albumin levels (Stewart et al, 1991; Pflüger et al, 1993; Liu et al, 1995). In our study, the only patient with grade IV haematological toxicity had

© Cancer Research Campaign 1997

mildly impaired renal function and an increased fraction of unbound etoposide in plasma. The percentage unbound etoposide was 13%, whereas a mean of 5% was reported in subjects with normal serum bilirubin and albumin levels (Stewart et al, 1989).

The present study was not designed to prove the efficacy of oral etoposide phosphate at the chosen dose and schedule. No objective responses were seen, but the patients in the study were often heavily pretreated and only 12 were evaluable for tumour response.

Interest in the oral administration of etoposide phosphate was raised because oral etoposide showed promising results in second-line treatment of several malignancies (Hainsworth and Greco, 1995), but its incomplete and variable bioavailability is an important drawback. This is a disadvantage that etoposide shares with other commonly used oral anti-cancer drugs. Similar variation in bioavailability has been reported for melphalan and cyclophosphamide, whereas bioavailability of oral mercaptopurine was only 16% (Bosanquet and Gilby, 1982; Zimm et al, 1983; Wagner and Fenneberg, 1984). Improving the pharmacological properties of oral anti-cancer drugs is therefore an important goal in the development of these drugs. Oral administration of the prodrug etoposide phosphate instead of etoposide did not result in a clinically relevant improvement as despite a small significant increase in bioavailability, variability appeared to be unaltered.

### ACKNOWLEDGEMENTS

We are indebted to P Bouma, C Prins and EAM Slijfer of the Department of Pharmacy at the University Hospital Groningen for their expert technical assistance with the pharmacological analysis. Bristol-Myers Squibb, Wallingford CT, supplied the drugs and supported data management.

### REFERENCES

Bosanquet AG and Gilby ED (1982) Pharmacokinetics of oral and intravenous melphalan during routine treatment of multiple myeloma. *Eur J Cancer Clin Oncol* **18**: 355–362

Budman DR, Igwemezie LN, Kaul S, Behr J, Lichtman S, Schulman P, Vinciguerra V, Allen SL, Kolitz J, Hock K, Oneill K, Schacter L and Barbhaiya RH (1994) Phase I evaluation of a water-soluble etoposide prodrug, etoposide phosphate, given as a 5-minute infusion on days 1, 3, and 5 in patients with solid tumours. *J Clin Oncol* **12**: 1902–1909

Chabot GG, Armand J-P, Terret M, de Forni M, Abigerges D, Winograd B, Igwemezie L, Schacter L, Kaul S, Ropers J and Bonnay M (1996) Etoposide bioavailability after oral administration of the prodrug etoposide phosphate in cancer patients during a phase I study. *J Clin Oncol* **14**: 2020–2030

Cockroft DW and Gault MH (1976) Prediction of creatinine clearance from serum creatinine. *Nephron* **16**: 31–41

Fields SZ, Igwemezie LN, Kaul S, Schacter LP, Schilder RJ, Litam PP, Himpler BS, McAleer C, Wright J, Barbhaiya RH, Langer CJ and O'Dwyer P (1995) Phase I study of etoposide phosphate (Etopophos) as a 30-minute infusion on days 1, 3, and 5. *Clin Cancer Res* **1**: 105–111

Gibaldi M and Perrier D (1982) *Pharmacokinetics*, 2nd edn, pp. 409–416. Marcel Dekker: New York

Hainsworth JD and Greco FA (1995). Etoposide: Twenty years later. *Ann Oncol* **6**: 325–341

Hainsworth JD, Levitan N, Wampler GL, Belani CP, Seyedsadr M, Randolph J, Schacter L and Greco FA (1995) Phase II randomized study of cisplatin plus etoposide phosphate or etoposide in the treatment of small-cell lung cancer. *J Clin Oncol* **13**: 1436–1442

Hande KR, Krozely MG, Greco FA, Hainsworth JD and Johnson DH (1993) Bioavailability of low-dose oral etoposide. *J Clin Oncol* **11**: 374–377

Harvey VJ, Slevin ML, Joel SP, Smythe MM, Johnston A and Wrigley PFM (1985) Variable bioavailability following repeated oral doses of etoposide. *Eur J Cancer Clin Oncol* **21**: 1315–1319

Hoskins PJ and Swenerton KD (1994) Oral etoposide is active against platinum-resistant epithelial ovarian cancer. *J Clin Oncol* **12**: 60–63

Liu B, Earl HM, Poole CJ, Dunn J and Kerr DJ (1995) Etoposide binding in cancer patients. *Cancer Chemother Pharmacol* **36**: 506–512

Martin M, Lluch A, Casado A, Santabarbara P, Androver E, Valverde JJ, Lopez-Martin JA, Rodriguez-Lescure A, Azagra P, Garcia-Conde J and Diaz-Rubio E (1994) Clinical activity of chronic oral etoposide in previously treated metastatic breast cancer. *J Clin Oncol* **12**: 986–991

Pflüger K-H, Hahn M, Holz J-B, Holz J-B, Schmidt, Köhl P, Fritsch H-W, Jungclas H and Haveman K (1993) Pharmacokinetics of etoposide: correlation of pharmacokinetic parameters with clinical conditions. *Cancer Chemother Pharmacol* **31**: 350–356

Riegelman S and Collier P (1980) The application of statistical moment theory to the evaluation of in vivo dissolution time and adsorption time. *J Pharmacokinet Biopharm* **8**: 509–534

Saulnier MG, Langley DR, Kadow JF, Senter PD, Knipe JO, Tun MM, Vyas DM and Doyle TW (1994) Synthesis of etoposide phosphate, BMY-40481: a water-soluble clinically active prodrug of etoposide. *Bioorg Med Chem Lett* **4**: 2567–2572

Sessa C, Zuchetti M, Cerny T, Pagani O, Cavalli F, De Fusco M, De Jong J, Gentili D, McDaniel C, Prins C, Schacter L, Winograd B and D'Incalci M (1995) Phase I clinical and pharmacokinetic study of oral etoposide phosphate. *J Clin Oncol* **13**: 200–209

Slevin ML, Clark PI, Joel SP, Malik S, Osborne RJ, Gregory WM, Lowe DG, Reznek RH and Wrigley PFM (1989*a*) A randomized trial to evaluate the effect of schedule on the activity of etoposide in small-cell lung cancer. *J Clin Oncol* **7**: 1333–1340

Slevin ML, Joel SP and Whomsley R (1989*b*) The effect of dose on the bioavailability of oral etoposide: confirmation of a clinically relevant observation. *Cancer Chemother Pharmacol* **24**: 329–331

Stewart CF, Arbuck SG, Pieper JA and Evans WE (1989) Altered protein binding in patients with cancer. *Clin Pharmacol Ther* **45**: 49–55

Stewart CF, Fleming RA, Arbuck SG and Evans WE (1990) Prospective evaluation of a model for predicting etoposide plasma protein binding in cancer patients. *Cancer Res* **50**: 6854–6856

Stewart CF, Arbuck SG, Fleming RA and Evans WE (1991) Relation of systemic exposure to unbound etoposide and haematologic toxicity. *Clin Pharmacol Ther* **50**: 385–393

Wagner T and Fenneberg K (1984) Bioavailability of cyclophosphamide from oral formulations. *Eur J Clin Pharmacol* **26**: 269–270

World Health Organization (1979) *Handbook for Reporting Results of Cancer Treatment*. Nijhoff: The Hague, The Netherlands

Zimm S, Collins JM, Riccardi R, O'Neill D, Narang PK, Chabner B and Poplack DG (1983) Variable bioavailability of oral mercaptopurine. Is maintenance chemotherapy in acute lymphoblastic leukemia being optimally delivered? *N Engl J Med* **308**: 1005–1009

*© Cancer Research Campaign 1997*